# Impact of protocolized postarrest care with targeted temperature management on the outcomes of cardiac arrest survivors without temperature management

**DOI:** 10.1080/07853890.2021.2016941

**Published:** 2021-12-22

**Authors:** Dean-An Ling, Chien-Hua Huang, Wen-Jone Chen, Po-Ya Chuang, Wei-Tien Chang, Chih-Wei Sung, Wei-Ting Chen, Hooi-Nee Ong, Min-Shan Tsai

**Affiliations:** a Department of Emergency Medicine, National Taiwan University Medical College and Hospital, Taipei, Taiwan; b School of Health Care Administration, Taipei Medical University, Taipei, Taiwan; c Department of Emergency Medicine, National Taiwan University Hospital Hsin-Chu Branch, Hsinchu, Taiwan

**Keywords:** Cardiac arrest, postarrest care, protocolized approach, targeted temperature management, neurological outcomes

## Abstract

**Introduction:**

Protocolized postarrest care that includes targeted temperature management (TTM) improves survival and neurological outcomes in cardiac arrest survivors. Whether the accumulated experience regarding the use of the protocolized approach also benefits patients who did not undergo TTM has yet to be investigated.

**Methods:**

Adults (≥18 years old) with nontraumatic cardiac arrest and who survived to intensive care unit (ICU) admission were retrospectively recruited from a single tertiary medical centre from 2006 to 2009 and 2011 to 2017. Patients were excluded if they had traumatic injuries, were pregnant, did not survive to ICU admission, regained clear consciousness within 3 h after the return of spontaneous circulation, or underwent TTM. The sum of TTM cases since 2006 and before the cardiac arrest of each enrolled patient was used as a substitute index for the amount of experience accumulated from the use of protocolized TTM care.

**Results:**

In total, 802 non-TTM patients were enrolled in the final analysis. The rate of survival to hospital discharge increased from 25.9% in 2006 to 33.3% in 2017. Regarding neurological recovery at hospital discharge, the incidence of favourable neurological function (cerebral performance category: 1 or 2) increased from 10.3% in 2006 to 23.5% in 2017. A multiple logistic regression indicated a significant association between the cumulative TTM case numbers and neurological outcomes in patients who did not receive TTM.

**Conclusions:**

The improvement of neurological outcomes in adult nontraumatic cardiac arrest survivors who did not receive TTM was associated with the cumulative number of cases receiving protocolized TTM care. In the era of TTM, the use of only historical control data might lead to bias, which is caused by overlooking the influence of a more refined protocolized postarrest care that includes TTM.

**KEY MESSAGE**
- The cumulative number of cases receiving protocolized TTM care, which we used as a substitute index for the amount of experience accumulated from the use of protocolized postarrest care that includes TTM, was associated with the improvement of neurological outcomes in adult nontraumatic cardiac arrest survivors who did not receive TTM.

## Introduction

Sudden cardiac death is a leading cause of morbidity and mortality. Sudden cardiac death’s annual incidence is approximately 15–100 per 100,000 in the general population, varying based on region, definition, and method of reporting [1]. The estimated incidence of sudden cardiac death in Taiwan is approximately 21,000–22,000 per year. Despite efforts to provide high-quality cardio-pulmonary-cerebral resuscitation and postarrest care, the outcome of sudden cardiac death remains poor [2], and the costs of resuscitation and postarrest care notably affect health care systems. Postarrest care is a key factor for maximizing survival and neurological outcomes in patients with cardiac arrest.

A protocolized bundle care approach of postarrest care, including targeted temperature management (TTM), improves the quality of care and the outcomes of cardiac arrest survivors [3]. The protocolized approach has been applied to several medical conditions, including resuscitation, sepsis, ventilator-associated pneumonia, and central line insertion, and has facilitated medical teams to provide more consistent care and overcome barriers [3–6]. For resuscitation, establishing a formal and structured emergency resuscitation protocol is beneficial in increasing the return of spontaneous circulation (ROSC) rate [4]. With regard to sepsis care, although a randomized trial did not reveal early goal-directed therapy (EGDT) to be beneficial in reducing all-cause mortality at 90 days, the improvement of quality of medical care along with the establishment of a protocol was proposed as a reason for similar outcomes in the EGDT and control groups [7]. TTM has been incorporated as part of postarrest care regardless of the initial rhythm. The optimal goal temperature has been evaluated in recent studies such as the TTM and TTM2 trials [8–12]. The refinement of protocolized postarrest care since 2002 might be one explanation for the comparability of outcomes between the hypothermia and normothermia group [11,12].

Despite the wide indications of TTM, comatose cardiac arrest survivors with time >12 h from the return of spontaneous circulation (ROSC), new intracranial haemorrhage, active major bleeding, unstable hemodynamic status despite inotropes, fatal arrhythmias, dementia or other cause of consciousness disturbance before arrest, terminal disease, and pregnancy are not suitable to undergo TTM [8,9]. These cardiac arrest survivors, although not receiving TTM, still undergo protocolized bundle care in intensive care units (ICUs), which involves ventilator adjustment, hemodynamic monitoring, neurological examinations, optimization of metabolic factors and electrolytes, seizure control, and cerebral images. Whether cardiac arrest survivors who do not undergo TTM also benefit from the bundle care approach remains unclear. Thus, in this study, we aimed to determine the influence of accumulated experience regarding protocolized postarrest care that includes TTM on the outcomes of cardiac arrest survivors without TTM.

## Methods

### Design and setting

This retrospective cohort study enrolled adults (≥18 years old) who survived a nontraumatic cardiac arrest in the emergency department of National Taiwan University Hospital (NTUH) at any period from 2006 to 2009 and 2011 to 2017. This study was approved by the Institutional Review Board of NTUH (IRB number: 201911017RINC), and the requirement for informed consent was waived. The study was performed in accordance with relevant guidelines and regulations.

### Clinical implication of TTM and establishment of postarrest care protocol

**Figure 1.**
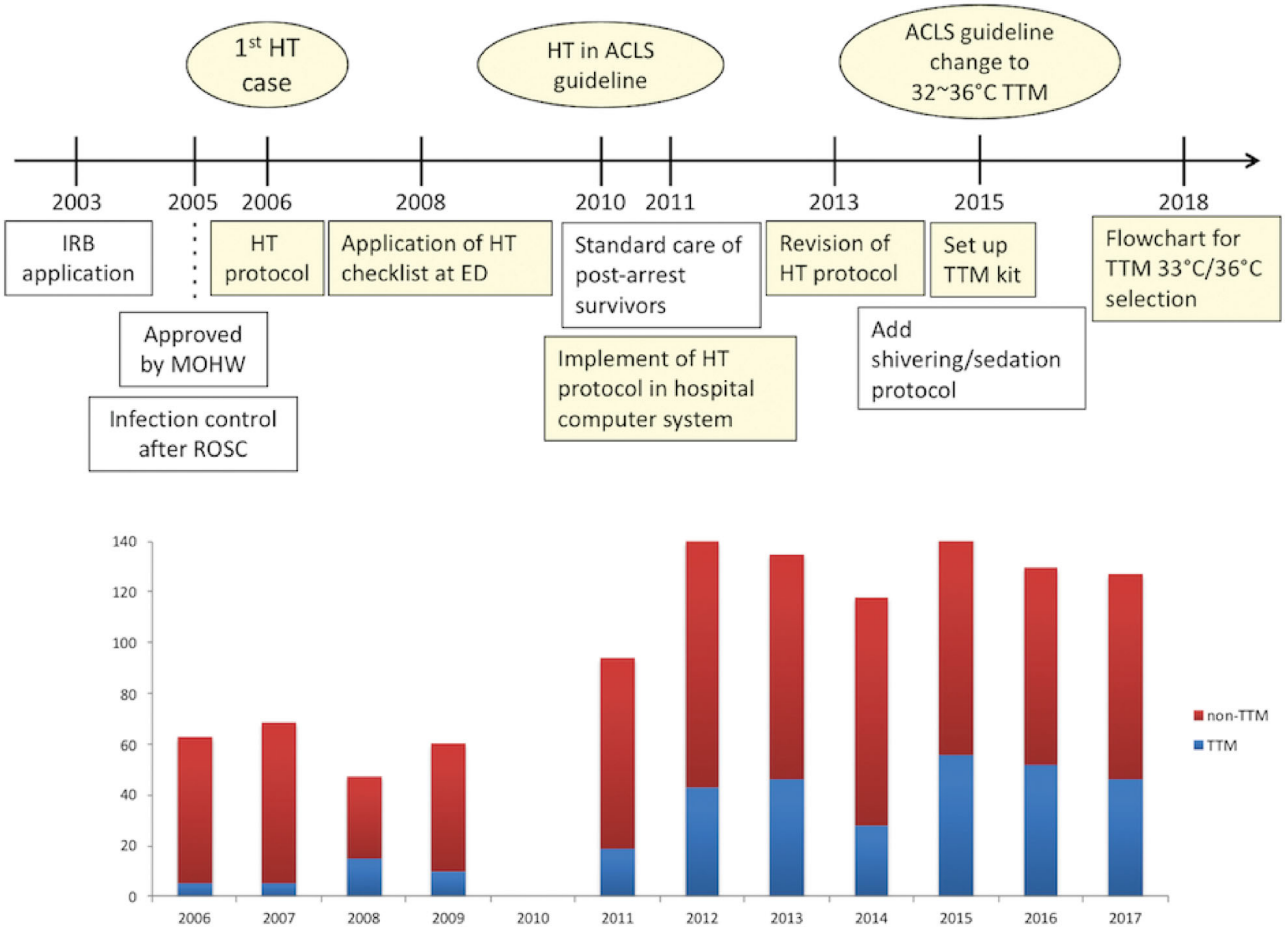
Development timeline of targeted temperature management protocol at National Taiwan University Hospital.

NTUH, a tertiary referral centre with approximately 100,000 emergency department visits per year, is located in Taipei City, Taiwan [13]. As the first hospital in Taiwan to introduce therapeutic hypothermia in unconscious cardiac arrest survivors, NTUH began to establish its protocol of bundle care for therapeutic hypothermia (which features brain computed tomography, hemodynamic monitors, and the optimisation of enteral nutrition, ventilator settings, blood sugar and electrolyte levels) since 2003. In 2005, the Taiwanese Ministry of Health and Welfare approved the application of intravascular cooling devices in cardiac arrest survivors, and, in 2006, therapeutic hypothermia was first applied to a cardiac arrest survivor. Therapeutic hypothermia was incorporated into the advanced cardiovascular life support (ACLS) guidelines in 2010 [3], and the hypothermia protocol was implemented in the NTUH computer system in 2011. After hospital staff acquired further evidence for and experience in therapeutic hypothermia, a revised hypothermia protocol, which involved the use of electroencephalography (EEG) readings, brain perfusion scans, the bispectral index, and cerebral oximeters, was established in 2013. In 2015, a TTM kit package was established to facilitate the TTM process and formulate the protocols of shivering and sedation. The ACLS guideline suggests a target temperature between 32 °C and 36 °C. The criteria and flowchart for the target temperature of 33 °C to 36 °C were established in 2018 (Figure 1). The current TTM protocol at the NTUH includes using cold saline and cooling devices with auto feedback to reduce patients’ body temperatures to the target temperature within 4–6 h after ROSC, to maintain the target temperature for 24 h, and to rewarm patients at the speed of 0.25 °C per hour until 36 °C is achieved. Temperature management is continued for another 24 h after rewarming to avoid fever. The checklist for postarrest care for cardiac arrest survivors at NTUH is included in the Supplementary Table 1.

**Table 1.** Baseline characteristics and cardiopulmonary resuscitation events of enrolled patients.

	Total		survivors			Favourable neurological outcome in survivors*		
Case number	*N* = 802		*N* = 228		*p*	*N* = 105		*p*
Age ≥ 65 (year-old)	488	60.8%	140	61.4%	0.839	50	47.6%	.003
Sex (Male)	495	61.7%	141	61.8%	0.964	65	61.9%	.967
Year					0.341			.003
2006	58	7.2%	15	6.6%		6	5.7%	
2007	64	8.0%	12	5.3%		6	5.7%	
2008	32	4.0%	9	3.9%		3	2.9%	
2009	50	6.2%	9	3.9%		5	4.8%	
2011	75	9.4%	18	7.9%		5	4.8%	
2012	97	12.1%	28	12.3%		6	5.7%	
2013	89	11.1%	33	14.5%		11	10.5%	
2014	90	11.2%	26	11.4%		10	9.5%	
2015	88	11.0%	27	11.8%		15	14.3%	
2016	78	9.7%	24	10.5%		19	18.1%	
2017	81	10.1%	27	11.8%		19	18.1%	
Comorbidities								
Diabetes mellitus	256	31.9%	77	33.8%	0.478	35	33.3%	.739
Hypertension	397	49.5%	124	54.4%	0.081	50	47.6%	.679
Coronary artery disease	226	28.2%	73	32.0%	0.128	36	34.3%	.136
Heart failure	70	8.7%	21	9.2%	0.760	9	8.6%	.951
Arrhythmia	88	11.0%	34	14.9%	0.024	17	16.2%	.066
COPD/asthma	72	9.0%	23	10.1%	0.488	7	6.7%	.374
Renal diseases	72	9.0%	24	10.5%	0.334	11	10.5%	.564
End-stage renal disease	64	8.0%	18	7.9%	0.955	9	8.6%	.810
Malignancy	211	26.3%	37	16.2%	<0.001	17	16.2%	.012
Witnessed collapse	622	77.6%	191	83.8%	0.008	91	86.7%	.016
Repeat CPR	160	20.0%	33	14.5%	0.014	13	12.4%	.037
Initial shockable rhythm	120	15.0%	63	27.6%	<0.001	42	40.0%	<.001
Adrenaline dosage ≥ 3mg	388	48.4%	79	34.6%	<0.001	30	28.6%	<.001
Cardiogenic arrest	313	39.0%	111	48.7%	<0.001	71	67.6%	<.001

The percentage of each characteristic and cardiopulmonary resuscitation event in total column were divided by total case numbers (*n* = 802). The percentage in survival column were divided by the case numbers of survivors (*n* = 228), and the percentage in favourable neurological outcome were divided by the case number of favourable neurological outcome in survivors (*n* = 105).

*Defined as Glasgow–Pittsburgh Cerebral Performance Category 1 or 2.

COPD: chronic obstructive pulmonary disease; CPR: Cardiopulmonary resuscitation; IHCA: in-hospital cardiac arrest; OHCA: out-of-hospital cardiac arrest.

### Patient enrolment

The number of patients who undergo TTM has stabilized since 2012 to approximately 45 patients annually, and, from 2006 to 2017, 325 cardiac arrest survivors underwent TTM (Figure 1). After excluding patients who had traumatic injuries, were pregnant, did not survive to ICU admission, regained clear consciousness within 3 h after ROSC, and all cardiac arrest survivors in 2010 (excluded due to the difficulty in accessing the information as the result of a transition in the health care information system in NTUH), we enrolled a total of 802 adult patients who had a nontraumatic cardiac arrest, survived to ICU admission, and did not receive TTM at any period during 2006–2009 and 2011–2017.

### Data collection

We collected the information on the following from medical records: baseline characteristics, pre-existing comorbidities, prehospital events, and in-hospital managements. Out-of-hospital cardiac arrest (OHCA) was defined the cessation of cardiac mechanical activity outside the hospital with a confirmed absence of any sign of circulation. In-hospital cardiac arrest (IHCA) was defined as the cessation of cardiac mechanical activity and confirmation without any sign of circulation after triage. Transferred cardiac arrest patients were defined as patients with cardiac arrest who gained ROSC after resuscitation at another hospital and who were transferred to NTUH for postarrest care. An initial shockable rhythm was considered the initial rhythm recorded as ventricular fibrillation or ventricular tachycardia. Adrenaline dosage was recorded as the sum of the dosages in both prehospital and hospital settings. Repeat cardiopulmonary resuscitation (CPR) was defined as a recurrent cardiac arrest event within 1 h after ROSC. A cardiogenic arrest was coded when the cause of arrest was assumed to be ischaemic heart disease, structural heart disease, heart failure, aortic dissection, or arrhythmia without electrolyte imbalance. Emergent coronary angiography was considered when being performed within 24 h after ROSC. We used the sum of TTM cases before cardiac arrest of each enrolled patient since 2006 as a substitute index for the accumulated experience regarding protocolized TTM care. Neurological outcome was defined as favourable if Glasgow–Pittsburgh Cerebral Performance Category 1 (*good performance*) or 2 (*moderate disability*) was reached.

### Statistical analysis

Binomial variables are presented in terms of number of cases (percentage) and were analysed using a chi-square or Fisher exact test. Multiple logistic regressions were used to determine the association between the cumulative number of TTM cases and outcomes, including survival and neurological outcomes. The variables used in multiple logistic regressions were those that were significant (*p* < .1) in univariate analyses. A *p*-value of <05 indicated statistical significance. Data were saved in a Microsoft Excel database and then analysed in Statistical Package for Social Sciences Statistics 21.0 (IBM, Chicago, IL, USA).

## Results

Among 802 non-TTM patients who survived to ICU admission, 228 patients survived to hospital discharge (28.4%) and 105 had favourable neurological outcomes (13.1%). The baseline demographic and clinical characteristics of the patients are presented in Table 1. The group of patients who survived to hospital discharge had a higher incidence of arrhythmia (*p* = .024) and a lower incidence of malignancies (*p* < .001) than the non-survivors did. With regard to resuscitation factors, the survivors had a greater incidence of witnessed collapse (*p* < .001), initial shockable rhythms (*p* < .001), and cardiogenic arrests (*p* < .001), lower incidence of adrenaline dosage > 3 mg (*p* < .001), and repeat CPR (*p* = .014) than the non-survivors. Survivors with favourable neurological outcomes were more likely to be >65 years old compared with those with poor outcomes (*p* = .003). The incidence of favourable neurological outcomes significantly increased with the year of enrolment (*p* = .003). The comparison of comorbidities and resuscitation factors between survivors with favourable versus poor outcomes was similar to that between survivors versus non-survivors, except there was no significant difference in the incidence of arrythmia.

**Figure 2.**
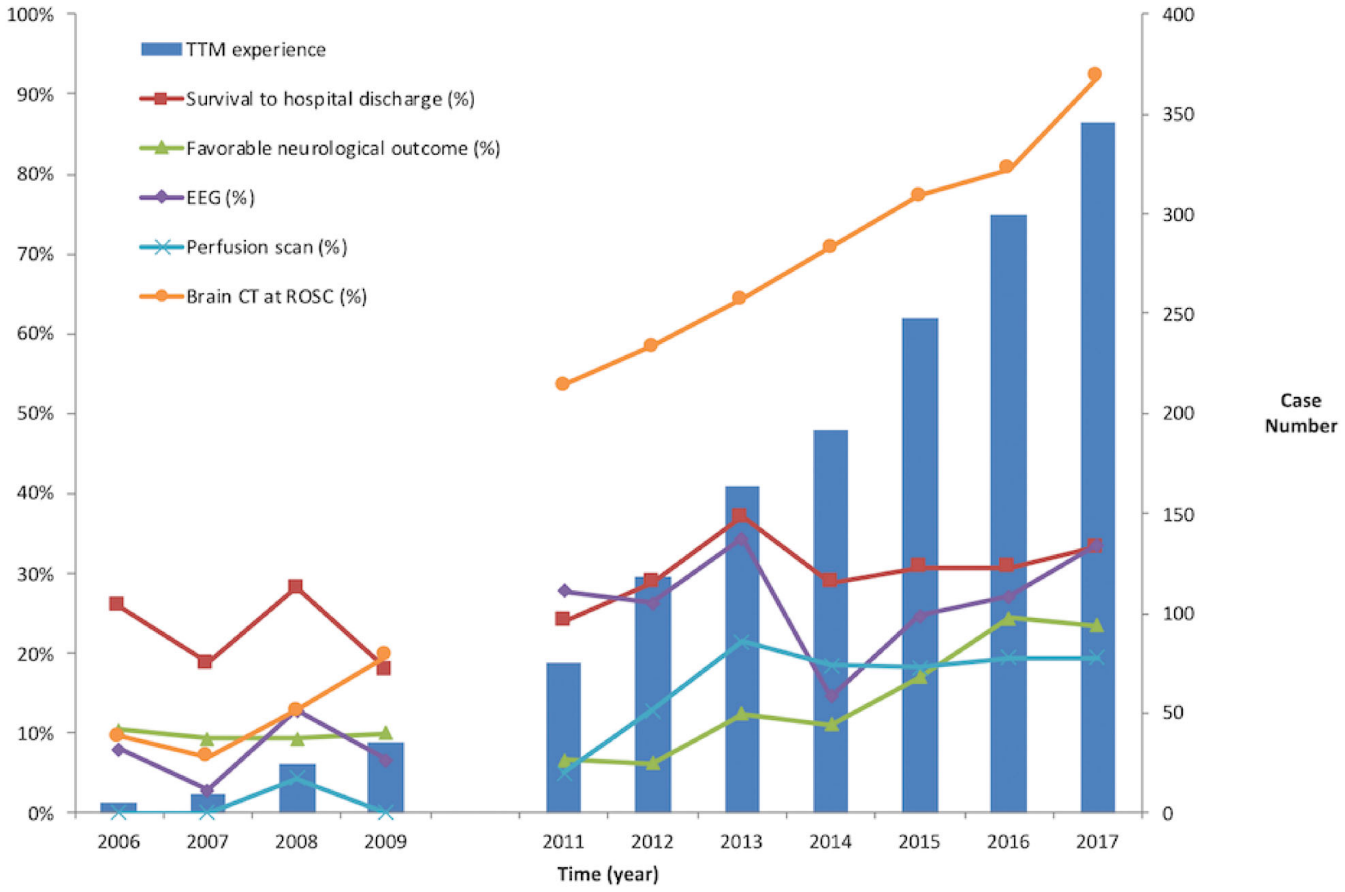
Annual changes in survival and neurological outcomes in cardiac survivors without TTM along with neuroprognostic examinations in all cardiac arrest survivors. CT: brain computed tomography; EEG: electroencephalography; ROSC: return of spontaneous circulation; TTM: targeted temperature management.

The trends of these variables over time are illustrated in Table 2. The rates of survival to hospital discharge increased from 25.9% in 2006 to 33.3% in 2017. With regard to neurological recovery at hospital discharge, the incidence of favourable neurological outcome increased from 10.3% in 2006 to 23.5% in 2017. The trends for survival and neurological outcomes, neuroprognostic examinations, and the accumulation of experience regarding TTM each year are presented in Figure 2. The trends of survival and neurological outcomes of TTM patients during the same period were presented in Supplementary Table 2. The proportion of cardiac arrest survivors, including both TTM and non-TTM patients, who underwent brain computed tomography (CT) at ROSC, EEG, and perfusion scan during postarrest care increased from 9.5%, 7.9%, and 0% in 2006 to 92.3%, 33.5%, and 19.4% in 2017, respectively. For non-TTM patients, the rates of undergoing brain CT after ROSC, EEG, and perfusion scan increased from 2%, 0%, and 0% in 2006 to 94%, 26%, and 11% in 2017, as displayed in Table 2. The multiple logistic regressions indicated a significant association between the cumulative TTM case numbers and favourable neurological outcomes (adjusted odds ratio = 1.003, 95% CI, 1.001–1.005; *p* = .008; Table 3) in the non-TTM patients, but they revealed no difference in survival to hospital discharge (adjusted odds ratio = 1.001, 95% CI, 0.999 − 1.002; *p* = .487; Table 4).

**Table 2.** The trend of baseline characteristics, CPR events and postarrest managements of patients without TTM over time.

	All	2006	2007	2008	2009	2011	2012	2013	2014	2015	2016	2017	*p*
Cardiac arrest survivors	1127	63	69	47	60	94	140	135	118	144	130	127	
Without TTM	802	58	64	32	50	75	97	89	90	88	78	81	
Accumulated TTM experience		5	10	25	35	75	118	164	192	248	299	346	
Characteristics													
Age ≥ 65 (year-old)	488 (61%)	42 (72%)	44 (69%)	23 (72%)	34 (68%)	43 (57%)	57 (59%)	48 (54%)	49 (54%)	56 (64%)	45 (58%)	47 (58%)	.258
Sex (Male)	495 (62%)	32 (55%)	36 (56%)	19 (59%)	25 (50%)	49 (65%)	64 (66%)	52 (58%)	63 (70%)	52 (59%)	47 (60%)	56 (69%)	.339
Comorbidities													
Diabetes mellitus	256 (32%)	20 (34%)	24 (38%)	14 (44%)	20 (40%)	19 (25%)	34 (35%)	31 (35%)	26 (29%)	34 (39%)	17 (22%)	17 (21%)	.068
Hypertension	397 (50%)	29 (50%)	33 (52%)	17 (53%)	27 (54%)	36 (48%)	47 (48%)	43 (48%)	41 (46%)	44 (50%)	41 (53%)	39 (48%)	.998
Coronary artery disease	226 (28%)	15 (26%)	16 (25%)	9 (28%)	14 (28%)	19 (25%)	34 (35%)	20 (22%)	27 (30%)	23 (26%)	25 (32%)	24 (30%)	.858
Heart failure	70 (9%)	8 (14%)	6 (9%)	1 (3%)	2 (4%)	9 (12%)	11 (11%)	4 (4%)	8 (9%)	7 (8%)	11 (14%)	3 (4%)	.179
Arrhythmia	88 (11%)	6 (10%)	6 (9%)	3 (9%)	3 (6%)	8 (11%)	7 (7%)	12 (13%)	10 (11%)	11 (13%)	11 (14%)	11 (14%)	.886
COPD/asthma	72 (9%)	9 (16%)	11 (17%)	2 (6%)	8 (16%)	7 (9%)	7 (7%)	8 (9%)	3 (3%)	9 (10%)	6 (8%)	2 (2%)	.029
Renal diseases	72 (9%)	6 (10%)	1 (2%)	3 (9%)	3 (6%)	6 (8%)	11 (11%)	10 (11%)	11 (12%)	10 (11%)	6 (8%)	5 (6%)	.532
End-stage renal disease	64 (8%)	3 (5%)	4 (6%)	2 (6%)	5 (10%)	7 (9%)	3 (3%)	14 (16%)	4 (4%)	8 (9%)	9 (12%)	5 (6%)	.122
Malignancy	211 (26%)	13 (22%)	14 (22%)	7 (22%)	10 (20%)	18 (24%)	23 (24%)	23 (26%)	26 (29%)	27 (31%)	22 (28%)	28 (35%)	.708
CPR events													
Witnesses collapse	622 (78%)	36 (62%)	39 (61%)	22 (69%)	31 (62%)	50 (67%)	74 (76%)	77 (87%)	73 (81%)	75 (85%)	73 (94%)	72 (89%)	<.001
Repeat CPR	160 (20%)	12 (21%)	19 (30%)	6 (19%)	16 (32%)	12 (16%)	26 (27%)	14 (16%)	12 (13%)	18 (20%)	12 (15%)	13 (16%)	.069
Initial shockable rhythm	120 (15%)	4 (7%)	7 (11%)	4 (13%)	2 (4%)	7 (9%)	12 (12%)	16 (18%)	21 (23%)	9 (10%)	16 (21%)	22 (27%)	<.001
Adrenaline dosage ≥ 3 mg	388 (48%)	28 (48%)	34 (52%)	18 (56%)	28 (56%)	32 (43%)	44 (45%)	44 (49%)	48 (53%)	38 (43%)	38 (49%)	37 (46%)	.842
CPR duration > 10 min	525 (65%)	39 (67%)	48 (75%)	22 (69%)	43 (86%)	42 (56%)	58 (60%)	65 (73%)	55 (61%)	57 (65%)	43 (55%)	53 (65%)	.010
Cardiogenic arrest	313 (39%)	12 (21%)	24 (38%)	13 (41%)	14 (28%)	28 (37%)	29 (30%)	38 (43%)	42 (47%)	38 (43%)	35 (45%)	40 (49%)	.013
Postarrest managements													
Brain CT at ROSC	401 (50%)	1 (2%)	1 (2%)	1 (3%)	2 (4%)	35 (47%)	47 (48%)	50 (56%)	62 (69%)	63 (72%)	63 (81%)	76 (94%)	<.001
EEG on post-ROSC Day 7	98 (12%)	1 (2%)	1 (2%)	2 (6%)	0 (0%)	12 (16%)	13 (13%)	24 (27%)	6 (7%)	7 (8%)	11 (14%)	21 (26%)	<.001
Perfusion scan on post-ROSC Day 7	41 (5%)	0 (0%)	0 (0%)	0 (0%)	0 (0%)	0 (0%)	4 (4%)	8 (9%)	9 (10%)	7 (8%)	4 (5%)	9 (11%)	.001
Echocardiogram at ROSC	287 (36%)	1 (2%)	1 (2%)	1 (3%)	0 (0%)	34 (45%)	45 (46%)	41 (46%)	44 (49%)	43 (49%)	39 (50%)	38 (47%)	<.001
Emergent CAG	109 (14%)	0 (0%)	0 (0%)	0 (0%)	0 (0%)	9 (12%)	11 (11%)	19 (21%)	19 (21%)	14 (16%)	15 (19%)	22 (27%)	<.001
ECMO	153 (19%)	9 (16%)	12 (19%)	14 (44%)	10 (20%)	12 (16%)	9 (9%)	19 (21%)	24 (27%)	11 (13%)	16 (21%)	17 (21%)	.004

The percentage of each baseline characteristic, CPR event and postarrest management were divided by the patient without TTM in each year.

CAG: coronary angiography; COPD: chronic obstructive pulmonary disease; CPR: Cardiopulmonary resuscitation; CT: computed tomography; ECMO: extracorporeal membrane oxygenation; EEG: electroencephalography; IHCA: in-hospital cardiac arrest; OHCA: out-of-hospital cardiac arrest; PCI: percutaneous coronary intervention; ROSC: return of spontaneous circulation; TTM: targeted temperature management.

**Table 3.** Association between accumulated targeted temperature management experience and favourable neurological outcome.

	Odds ratio (95% CI)	*p*
TTM experience	1.003 (1.001–1.005)	.008
Age ≥ 65 (year-old)	0.561 (0.352–0.893)	.015
Arrhythmia	1.027 (0.519–2.030)	.940
Malignancy	0.635 (0.344–1.172)	.146
Witnessed collapse	1.474 (0.770–2.822)	.242
Repeated CPR	0.444 (0.228–0.867)	.017
Initial shockable rhythm	2.868 (1.626–5.057)	<.001
Adrenaline dosage ≥3mg	0.218 (0.129–0.368)	<.001
Cardiogenic arrest	3.323 (1.908–5.789)	<.001

CPR: Cardiopulmonary resuscitation; IHCA: in-hospital cardiac arrest; OHCA: out-of-hospital cardiac arrest; TTM: targeted temperature management.

**Table 4.** Association between accumulated targeted temperature management experience and survival to hospital discharge.

	Odds ratio (95% CI)	*p*
TTM experience	1.001 (0.999–1.002)	.487
Age ≥ 65 (year-old)	1.102 (0.780–1.559)	.582
Arrhythmia	1.264 (0.760–2.103)	.367
Malignancy	0.422 (0.275–0.647)	<.001
Witnessed collapse	1.576 (1.023–2.428)	.039
Repeated CPR	0.548 (0.350–0.859)	.009
Initial shockable rhythm	3.188 (1.971–5.158)	<.001
Adrenaline dosag*e* ≥ 3mg	0.371 (0.261–0.526)	<.001
Cardiogenic arrest	1.216 (0.823–1.797)	.326

CPR: Cardiopulmonary resuscitation; IHCA: in-hospital cardiac arrest; OHCA: out-of-hospital cardiac arrest; TTM: targeted temperature management.

## Discussion

This study demonstrated that the favourable neurological outcomes of non-TTM cardiac arrest survivors improved with the increase in cumulative number of TTM cases, which were used as a substitute index for the accumulated experience regarding protocolized TTM care. The underlying characteristics and resuscitation factors were adjusted. Studies have verified that in experienced regionalized centres, clinicians can improve the outcomes of cardiac arrest survivors by applying multidisciplinary postarrest treatments, including TTM, early coronary revascularization, and systematic and brain-oriented intensive care [14,15]. However, the evidence of an association between outcome improvement and the evolution of the postarrest care protocol and treatment experience through time, even in patients who did not receive TTM or emergent coronary angiography, is lacking. This study demonstrated the improvement of neurological outcomes in cardiac arrest survivors without TTM in parallel with the refinement of postarrest care in NTUH with an emphasis on neurological care.

Protocolized postarrest care together with TTM reportedly improves neurological outcomes in cardiac arrest survivors [3]. In the TTM era, cardiac arrest survivors who did not undergo TTM due to contraindications, financial issues, or a decision made by their family still received protocolized postarrest care in the ICU. The bundle care included hemodynamic monitoring, ventilator adjustment, metabolic optimisation, seizure control, and adequate sedation. The application of EEG, brain CT, perfusion scans, bispectral index monitors, and cerebral oximeters has refined the provision of neurological care. The introduction of neuromonitoring has facilitated the early detection and management of seizure and intracranial haemorrhage. The proportion of cardiac arrest survivors who underwent neuroprognostic examinations increased over time. Brain CT had the highest rate of increase whereas those for EEG and perfusion plateaued after 2013. This might have been influenced by the timing of the examinations because some patients passed away before receiving EEG, and perfusion is scheduled for approximately the seventh day after ROSC. Although hemodynamic monitoring, respiratory support, and metabolic optimisation are already part of standard care in ICU, neurophysiological and neuroimage examinations are not. This might explain why an improved protocolized approach proved to be more beneficial for neurological recovery than for survival in this study. The survival rate of current study was 28.4%, which was higher than most previous reports of emergency medical services treated OHCA across the world (3.1–20.4%) [16]. Different from the International Liaison Committee on Resuscitation (ILCOR) report, we enrolled cardiac arrest survivors who survived to ICU admission, including both OHCA and IHCA patients, and excluded traumatic arrests. The patients were all from a tertiary centre. These may all influence the survival rate. As a comparison, the multi-centre TIMECARD registry for TTM patients in Taiwan between January 2014 and September 2019 showed the survival rate of 41.9%, and favourable neurological outcome of 21.7% [17].

The in-hospital mortality rate has reduced over time in sepsis care for ICU patients with severe sepsis and septic shock, even in patients who did not receive EGDT [7]. Likewise, this study demonstrated that protocolized postarrest care including TTM is associated with the improvement of outcomes in cardiac arrest survivors without TTM. For studies comparing the effects of treatment, the improvement of care quality over time must be considered to avoid possible bias. As indicated in Table 2, resuscitation factors changed over time. The promotion of public awareness and faster emergency medical service response reportedly increases the percentage of witnessed collapse and the initial shockable rhythm. These might also contribute to improved prognosis. However, after these variables were adjusted for in multiple logistic regression, the cumulative TTM case numbers that were used as a substitute index for the accumulated experience regarding protocolized TTM care was still significantly associated with favourable neurological outcome. In recent TTM trials, hypothermia and normothermia groups had comparable outcomes [11], conflicting with results from previous hypothermia studies [9]. The refinement of protocolized postarrest care, which improved the outcomes of hypothermia, normothermia, and even non-TTM cardiac arrest survivors, may have contributed to these results.

This study had several limitations. First, owing to a transition in the health information system at NTUH, not all information on adult nontraumatic cardiac arrest survivors in 2010 was available. Therefore, cardiac arrest survivors in 2010 were excluded from the analysis. Second, the effect of the evolving management for cardiac arrest patients over time could not be eliminated as a result of these changes being part of the protocolized postarrest care. The TTM experience might be a surrogate for advancement in care over time which contributes to the improved outcomes. The improvement of pre-hospital emergency care and the refinement of ACLS increased the successful rate of resuscitation, therefore drive the emphasis on the postarrest care including TTM. The effect of chain of survival including prehospital emergency care, ACLS teamwork and advancement, and postarrest care, on cardiac arrest survivors is hard to isolate from each other. Along with the application of TTM, lots of efforts had been put on establishing protocols and refining the postarrest care to ensure the quality. It is the reason why we chose TTM experience as a substitute index for the accumulated experience regarding protocolized postarrest care. Third, selection bias was unavoidable due to the retrospective design of the study, and unidentified confounding factors may be present. Future prospective randomized trial is needed to provide more evidence. Finally, the reasons for patients not receiving TTM were not recorded. The socioeconomic status of these patients, which might affect willingness to undergo TTM, was also not considered in this study. However, standard ICU and postarrest care are covered by National Health Insurance in Taiwan, and TTM was incorporated into the coverage in October 2015. Future studies to explore the epidemiology and neuroprognosis of non-TTM patients might provide more insights to the non-TTM cohorts.

## Conclusions

The improvement of neurological outcome in adult nontraumatic cardiac arrest survivors who did not receive TTM was associated with the cumulative number of TTM cases. In the TTM era, the use of only historical control data might lead to bias caused by the overlooking of the influence from refinements in protocolized postarrest care that includes TTM.

## Supplementary Material

Supplemental Material
