# Supplementary material for: Impact of protocolized postarrest care with targeted temperature management on the outcomes of cardiac arrest survivors without temperature management
Source: Ann Med. 2021 Dec 22;54(1):63–70. doi: 10.1080/07853890.2021.2016941 (PMC8725984; doi:10.1080/07853890.2021.2016941)
Supplement: Supplemental Material [file IANN_A_2016941_SM8737.docx]

**Supplementary material**

Supplementary Table 1. Postarrest care checklist for cardiac arrest survivors at National Taiwan University Hospital.

| **Induction phase (4-6hr)** | **Maintenance phase (24hr)** | **Rewarming phase (12-16hr)** | **Normothermia phase (24hr)** |
| --- | --- | --- | --- |
| Record body temperature every hour  Record shivering score every hour (keep BSAS=0)  Record RASS every hour (Keep RASS -2~-3)  Check brain CT  Check ECG  Give 4℃ cold Normal Saline  Choose cooling device  Set ventilator humidifier at 33℃  On CVC, PiCCO  Keep SBP > 90 mmHg  Record cardiac output  Use BIS or Cerebral oximeter  Keep NPO and give D5W IVF, Check FSBS regularly, keep < 200 mg/dl  Use sedation, analgesic medications, and muscle relaxant  Check CBC, biochemistry profiles, cardiac enzyme, coagulation profiles | Record body temperature every hour  Record shivering score every hour (keep BSAS=0)  Record RASS every hour (Keep RASS -2~ -3)  Check ECG (6 hours after reach 33℃)  Keep NPO and give D5W IVF, Check FSBS regularly, keep < 200 mg/dl  Keep SBP > 90 mmHg  Record cardiac output regularly  Use BIS or Cerebral oximeter continuously  Evaluation the use of sedation, analgesic medications, and muscle relaxant  Check CBC, biochemistry profiles, cardiac enzyme, coagulation profiles regularly | Rewarm 0.25℃ every hour  Record body temperature every hour  Record shivering score every hour (keep BSAS=0)  Record RASS every hour (Keep RASS -1~ -2)  Check ECG (when reach 36.5℃)  Set ventilator humidifier at 33℃  Keep NPO and give D5W IVF, Check FSBS regularly, keep < 200 mg/dl  Keep SBP > 90 mmHg  Record cardiac output regularly  Use BIS or Cerebral oximeter continuously  Reduce the dose of muscle relaxant if rewarming to 34.5℃  Taper sedation if rewarming to 35℃  Check CBC, biochemistry profiles, cardiac enzyme, coagulation profiles regularly | Try water or D5W from nasogastric tube, Check FSBS regularly, keep < 200 mg/dl  Record cardiac output regularly  Remove BIS or Cerebral oximeter  Discontinue muscle relaxant  Keep body temperature <36.5℃ |

BSAS: Bedside Shivering Assessment Scale; RASS: Richmond Agitation-Sedation Scale; CT: Computed Tomography; ECG: Electrocardiography; CVC: central venous catheter; PiCCO: pulse-induced contour cardiac output; SBP: systolic blood pressure; BIS: Bispectral index; NPO: nil per os (nothing by mouth); D5W: 5% Glucose; IVF: intravenous fluid; FSBS: finger-stick blood sugar; CBC: complete blood count

Supplementary Table 2. The trends of survival and neurological outcomes over time in cardiac arrest survivors with targeted temperature management.

| Year | Total | Survival to hospital discharge | | | | Neurological outcome | | | |
| --- | --- | --- | --- | --- | --- | --- | --- | --- | --- |
|  |  | Survivors | | Non-survivors | | Favorable | | Poor | |
| 2006 | 5 | 3 | 60.0% | 2 | 40.0% | 1 | 20.0% | 4 | 80.0% |
| 2007 | 5 | 1 | 20.0% | 4 | 80.0% | 0 | 0.0% | 5 | 100.0% |
| 2008 | 15 | 7 | 46.7% | 8 | 53.3% | 2 | 13.3% | 13 | 86.7% |
| 2009 | 10 | 6 | 60.0% | 4 | 40.0% | 1 | 10.0% | 9 | 90.0% |
| 2011 | 19 | 13 | 68.4% | 6 | 31.6% | 3 | 15.8% | 16 | 84.2% |
| 2012 | 43 | 22 | 51.2% | 21 | 48.8% | 7 | 16.3% | 36 | 83.7% |
| 2013 | 46 | 22 | 47.8% | 24 | 52.2% | 12 | 26.1% | 34 | 73.9% |
| 2014 | 28 | 18 | 64.3% | 10 | 35.7% | 10 | 35.7% | 18 | 64.3% |
| 2015 | 56 | 25 | 44.6% | 31 | 55.4% | 13 | 23.2% | 43 | 76.8% |
| 2016 | 52 | 23 | 44.2% | 29 | 55.8% | 16 | 30.8% | 36 | 69.2% |
| 2017 | 46 | 19 | 41.3% | 27 | 58.7% | 15 | 32.6% | 31 | 67.4% |
